# Scurvy-Rickets and Civilisation

**Published:** 1900-01-06

**Authors:** 


### SCURVY-RICKETS AND CIVILISATION.

IN a clinical lecture recently delivered at St. Mary's Hospital, Mr. Edmund Owen pointed out that the diet which produces rickets is the same as that which produces scurvy, and that both diseases are in the great majority of cases the result of replacing natural "living" foods by the various patent preparations which civilisation has introduced into the nursery. Rickets is generally regarded as especially a disease of infancy, but that is probably because the dyspeptic child who has struggled through the perils of his cradle life is directly afterwards put upon a mixed diet, which is the saving of him. There seems, however, no reason why, if a fairly healthy boy of, say, six or seven years of age were put upon a typical scorbutic diet, the ends of his bones should not swell and keep on swelling just as they do in cradle life. And much the same may be said of scurvy. There is, indeed, no boundary between the two, the rickets and the scurvy of childhood; rickets gradually and imperceptibly passes into scurvy, and when one is told that an infant or a child is the subject of scurvy, one need not be told that he is rachitic also. It might be thought that scurvy-rickets would be more apt to be found amongst the children of the poor than of the rich, but, as a matter of fact, the reverse is the case. If the poor woman sees the advertisements of these patent foods (so-called) she pays no heed to them—their price has fortunately placed them beyond her reach. But the well-to-do mother takes a delight in buying them for her darlings. Mr. Owen says that of the many cases of scurvy-rickets that he has seen his impression is that the worst were those which had been fed upon the most extensively-advertised patent foods. But it may occur even in children fed upon fresh cow's milk if this be too largely diluted, with barley water for example. Some infants, again, become scorbutic from being continuously fed upon pancreatised milk, and it is much to be feared that the habit of scalding nursery milk, although done with the laudable object of freeing it from certain disease germs, may materially detract from its value as a food for hand-reared infants. In fact, artificiality of every kind is to be avoided in infant feeding, and although some artificial compound may sometimes appear to suit a child and relieve it from certain dyspeptic troubles, it is well never to continue long with any kind of artificial food. It often happens that both doctor and mother are so well pleased when they have at last discovered a food which apparently suits the baby that they are afraid to give it up when the dietetic crisis for which it was ordered has passed away, but by so doing the intercurrence of rickets and of scurvy is invited. It has to be remembered that in many cases the child of the poor man obtains a more liberal and certainly a more varied diet than the child of the rich one; and although no one would approve of some of the things that poor men's children are often allowed to eat, the fact that they are fed on what is going for their elders gives them access to many forms of anti-scorbutic food, especially in the form of fruit, from which the children of the rich are carefully debarred. Civilisation is certainly not an unmixed blessing, especially when in the rearing of infants it substitutes artificial for natural forms of food.

